# Items of General Interest

**Published:** 1916-08

**Authors:** 


### Items of General Interest.

The Royal Surgical Society of London has received a gift of a large sum of money to be offered as a prize to the inventor of the best artificial hand.

The annual meeting of the American Electrotherapeutic Association will be held in New York September 12th, 13th and 14th, with headquarters at the Hotel Martinique.

The International Health Commission of the Rockefeller Foundation hereby announces the change of its name to International Health Board of the Rockefeller Foundation.

Mr. Oliver Iselin has sent a check to the Red Cross to pay for goggles for use by the New York National Guard on duty on the Mexican border to protect them from the alkali dust and glare of the sun.

At a recent session of the Legislature of New York a bill was passed providing for physical training of not less than twenty minutes each day by all pupils eight years of age and over in the public and private schools in New York State.

---

Sir William Osler has sent word to a number of American surgeons that there are vacancies for 120 young American medical graduates in the military hospitals in London and its immediate neighborhood. The term of service is six months. There will be a small salary, and passage will be paid both ways.

---

The American Red Cross headquarters announce that a base hospital unit at the German Hospital in New York is being organized under the American Red Cross. Its director is Dr. Frederick Kammerer, who has just withdrawn from active service with the German army, and brings to his new task the very valuable knowledge gained at the German front in the European war zone.

---

The Cuban sanitary authorities have adopted quarantine restrictions against the United States children on account of the epidemic of infantile paralysis. In the future children arriving from American ports who have an abnormal temperature will be isolated until the trouble is diagnosed, while children in normal health will be kept under surveillance until the danger of developing infantile paralysis is past.

---

Professor Elie Metchnikoff, of Paris, France, died on July 15th in an apartment of the Pasteur Institute, Paris. He was born in 1845 in Kharkov, Russia, where he began his studies. These were continued in a Russian University and in Giessen and Munich until 1870, when he was appointed Professor of Zoology in Petrograd and Odessa. In 1882 he resigned, and while studying along the shores of the Mediterranean formulated his theory of inflammation and announced his discovery of the rôle of the leucocytes. Metchnikoff succeeded the founder of the Pasteur Institute as director in 1895, and began his study of longevity.

---

The China Medical Board of the Rockefeller Foundation announces that the trustees of Union Medical College in Pekin have appointed Dr. Franklin Chambers McLean Professor of Internal Medicine, a position which carries with it the headship of the college. Union Medical College is one of the principal institutions through which the board is working to improve medical and hospital conditions in China.

Dr. McLean is now on his way to the Orient, where he will study the work of the China Medical Board in Pekin and Shanghai.

To accept the new position Dr. McLean resigned as Assistant Resident Physician in the Hospital of the Rockefeller Institute in New York.

